# A Rare Case of Posttraumatic Bilateral BPPV Presentation

**DOI:** 10.1155/2021/8636676

**Published:** 2021-09-06

**Authors:** Sinisa Maslovara, Andro Kosec, Ivana Pajic Matic, Anamarija Sestak

**Affiliations:** ^1^Department of Otorhinolaryngology, National Memorial Hospital Vukovar, Vukovar, Croatia; ^2^Department of Otorhinolaryngology Neurosurgery and Radiology, Faculty of Dental Medicine and Health, “Josip Juraj Strossmayer University” of Osijek, Osijek, Croatia; ^3^Department of Otorhinolaryngology and Head and Neck Surgery, University of Zagreb School of Medicine, University Hospital Center “Sisters of Mercy”, Zagreb, Croatia; ^4^Department of ENT Head and Neck Surgery, General Hospital “Dr. Josip Benčević”, Slavonski Brod, Croatia; ^5^Department of Otorhinolaryngology and Head and Neck Surgery, Clinical Hospital Centre Osijek, Osijek, Croatia

## Abstract

A rare case of a 38-year-old female patient who developed benign paroxysmal positional vertigo (BPPV) three weeks after head trauma is presented. The disease manifested bilaterally, which is not uncommon posttraumatically, but in this case, it manifested itself as canalithiasis of the posterior canal on both sides and cupulolithiasis of the right lateral canal, which to our knowledge is a unique and, until now, unpublished case. The aim of this review is to point out the fact that, in such a complex multicanal and bilateral clinical presentation of BPPV, it is not sufficient to perform only positioning but also additional laboratory tests. With a good knowledge of the etiopathogenesis, pathophysiology and clinical forms of BPPV, we can, in most cases, make an accurate and precise diagnosis of the disease and carry out appropriate treatment.

## 1. Introduction

Benign paroxysmal positional vertigo (BPPV) is the most common cause of vestibular vertigo in adult population with a prevalence of 19% among other causes, while in the elderly population, this share climbs up to 50% with a lifetime prevalence of 2.4% [1–3]. The disease is caused by the displacement of otoliths from the otolithic macula of the utriculus, which then travels through the endolymph to one of the semicircular canals, with the posterior canal (PC) affected in the majority of cases. Simultaneous multicanal BPPV (mBPPV) and a bilateral BPPV (bBPPV) involvement are also possible. Head trauma is one of the most known causes of the disease (8.2–15%), while most cases are idiopathic [4].

After searching the PubMed and Google Scholar databases with the keywords “bilateral,” “multicanal,” and “BPPV,” a total of 3 case reports of simultaneous multicanal and bilateral BPPV were found. Of these 3 case reports, only one of them was associated with head trauma, but none of them observed the simultaneous occurrence of canalithiasis and cupulolithiasis on opposite sides [5–7].

What is significant in our case report is a description of the course of the disease and the treatment procedures for this rare form of BPPV, and we present for the first time a video recording of canalithiasis on both sides and cupulolithiasis on the right side.

This case report was approved by the Ethics Committee of National Memorial Hospital Vukovar, Croatia, ID: EP-5/2021. Written informed consent was obtained from the patient.

## 2. Case Presentation

**Figure 1:**
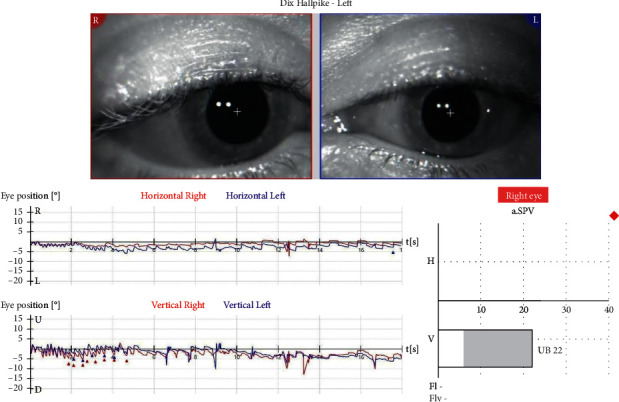
Left-sided Dix–Hallpike test performed during the initial videonystagmography (VNG) recorded upbeat nystagmus, of SPV 22°/s.

**Figure 2:**
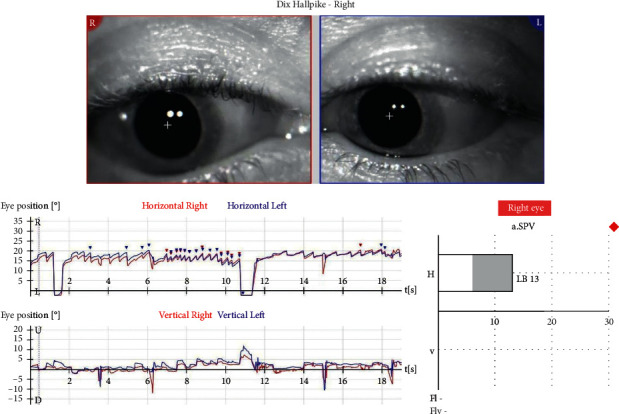
Right-sided Dix–Hallpike test performed during the initial VNG recorded horizontal ageotropic nystagmus of SPV 13°/s.

**Figure 3:**
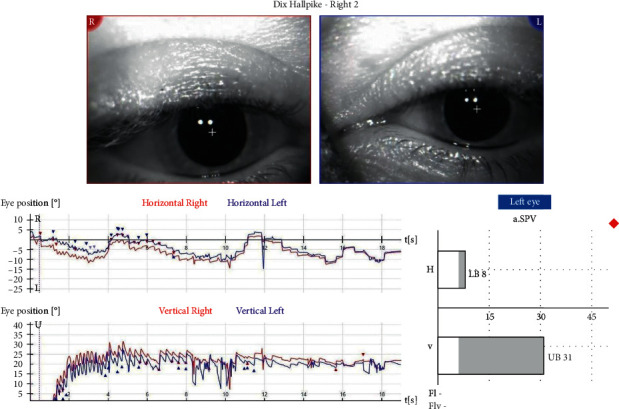
Right-sided Dix–Hallpike test performed during the follow-up exam a week after the initial VNG recorded geotropic vertical-rotatory PN lasting for 10 seconds with the upbeat directed vertical component of SPV 31°/sec and a torsion geotropic directed component of SPV 8°/sec.

We present a 38-year-old female patient that fell and hit the left side of her head during morning exercise in mid-December 2020. She underwent a neurological examination, MRI of the head and neck, without pathological findings. Three weeks later, she developed short episodes of rotational dizziness, occurring when lying on her back and turning to the left. A Dix–Hallpike test was bilaterally positive. On the left side, an upbeat nystagmus was observed, without latency, lasting for 5-6 seconds. When performing a right-sided test, an ageotropic horizontal nystagmus was observed after 4-5 seconds of latency. A supine-roll test was performed, and a horizontal ageotropic positioning nystagmus (PN) was induced bilaterally, more intense on the left. Although guidelines for the diagnosis of BPPV do not consider further vestibular testing necessary [8], we opted to undergo laboratory evaluation due to the complex presentation of the disease [9, 10]. The graphical recording of the left-sided Dix-Hallpike test performed as a part of videonystagmography (VNG) manifested an isolated upbeat nystagmus, measuring SPV 22°/s (Figure 1), while a right-sided test manifested a horizontal ageotropic nystagmus measuring SPV 13°/s (Figure 2). Video analysis manifested a discrete torsional, geotropically directed nystagmus in the left position (supplementary video 1). In video 2, the emergence of two different types of nystagmus can be observed, the first torsional nystagmus (from posterior canal BPPV) which was not immediately observed clinically or in the VNG graphical recording, followed by a purely horizontal nystagmus (from horizontal cupulolithiasis) (supplementary video 2). A diagnosis of the left-sided PC-BPPV canalithiasis and of the right-sided HC-BPPV cupulolithiasis was performed. Also, based on the torsional nystagmus that can be observed in video 2 (supplementary video 2), a diagnosis of right-sided PC-BPPV canalithiasis was also performed. Based on the clinical findings and VNG graphical recording, the corresponding left-sided Epley and right-sided barbecue-roll repositioning manoeuvres were performed. A week later, during the first follow-up exam, the patient complained about a mild dizziness when raising her head only. A follow-up left-sided Dix–Hallpike test was negative, while a right-sided Dix–Hallpike test resulted in a severe dizziness and an apogeotropic vertical-torsional nystagmus lasting for ten seconds, with a vertical component directed upwards, measuring 31°/sec, and with a torsional component measuring 8°/sec, directed to the right (geotropic) (Figure 3 and supplementary video 3). It was concluded that the left-sided PC canalithiasis was resolved, while the right-sided PC canalithiasis still remained. A right-sided Epley maneuver was performed. During the next weekly check-up, the patient reported a resolution of all symptoms. The follow-up Dix–Hallpike and supine-roll tests were subjectively and objectively negative.

## 3. Discussion

Even though the cause of BPPV remains unknown in most cases, in this case, it is likely associated with a head trauma experienced by the patient three weeks prior to the onset of vertigo. This is supported by a bilateral presentation, which is far more common in posttraumatic than in the idiopathic form of BPPV (25%: 2%) [11]. It is known that the otoconia debris (more than 90%) end up in the PC in most cases [12, 13]. It was long thought that it may be the only canal that can be affected. Later findings suggested that the lateral canal (HC-BPPV) can be affected in 1.9–24.7% of cases, often occurring as a conversion of BPPV after the PC repositioning procedures [14, 15]. A multicanal BPPV accounts for 12.5% of all BPPV cases. It is most often posttraumatic or associated with the middle- and inner-ear diseases, with a combination of PC and HC in the same labyrinth, while in the case of bBPPV, a bilateral PC involvement is the most common [16]. The prevalence of bBPPV is 2.9%, with the combination of PC and HC accounting for 12.5% of cases, which is about 0.3% of all BPPV cases [17]. In addition to the involvement of individual canals, a BPPV may be manifested in different clinical forms, depending on the location of the debris. The most common form by far is canalithiasis, with the placement of otolithic debris in the canal, while cupulolithiasis is much rarer but clinically more severe, with an otolith deposition adjacent to the cupula. Although canalithiasis and cupulolithiasis are reported as two separate clinical phenomena, it is not difficult to assume a simultaneous existence of a combination of these two entities, which has not been video documented so far. We induced the PN by a left-sided Dix–Hallpike test lasting for 5-6 seconds, with almost no latency, followed by nausea and an objective-rotational dizziness. Graphically, only an upbeat nystagmus was recorded. Most of these data, without a video analysis, would speak in favor of a left-sided PC cupulolithiasis. However, a short nystagmus duration, with a subsequent video analysis demonstrating a discrete torsional, clockwise-oriented PN component in addition to an upbeat nystagmus, favored a clinical form of canalithiasis. Though one of the features of cupulolithiasis, a short nystagmus latency is also possible in canalithiasis, when the otoconia debris are located close to the cupula. When performing the right-sided Dix–Hallpike test, after a short latency lasting for a few seconds, a horizontal ageotropic PN is observed clinically, lasting about 20 seconds, but video analysis showed the emergence of torsional nystagmus, followed by a purely horizontal nystagmus. While performing a supine-roll test, an apogeotropic horizontal PN was observed bilaterally, slightly more pronounced on the left, leading to the diagnosis of a right-sided HC cupulolithiasis. Some authors suggest performing the repositioning procedures only on a more symptomatic side and address the other side later on [18], but we have performed the bilateral repositioning procedures simultaneously with a favourable outcome.

Although PC is affected in the largest number of BPPV patients by far, a possible involvement of other canals should always be considered, and the Dix–Hallpike should be followed by a supine-roll test, especially when the patient complains about a position-related dizziness and when the Dix–Hallpike test is negative. Diagnosis should sometimes be supplemented by laboratory tests, especially in such a complex case of multicanal BPPV presentation.

## Data Availability

The data used to support the findings of this study are available from the corresponding author upon request.

## Supplementary Materials

Supplementary Materials Supplementary video 1: video recording of the initial left-sided Dix–Hallpike test. Supplementary video 2: video recording of the initial right-sided Dix–Hallpike test. Supplementary video 3: video recording of the follow-up right-sided Dix–Hallpike test.
