# Supplementary material for: A Rare Case of Posttraumatic Bilateral BPPV Presentation
Source: Case Rep Otolaryngol. 2021 Sep 6;2021:8636676. doi: 10.1155/2021/8636676 (PMC8440101; doi:10.1155/2021/8636676)
Supplement: Supplementary Materials — Supplementary video 1: video recording of the initial left-sided Dix–Hallpike test. Supplementary video 2: video recording of the initial right-sided Dix–Hallpike test. Supplementary video 3: video recording of the follow-up right-sided Dix–Hallpike test. [file 8636676.f1.docx]

Double-click to watch videos
